# Safety of Laparoscopic Entry Points in Patients With a History of Abdominal Surgery: A Research Article

**DOI:** 10.7759/cureus.47244

**Published:** 2023-10-18

**Authors:** Ender Güven, Mustafa Cengiz Dura, Hilal Aktürk, Hakan Güraslan

**Affiliations:** 1 Obstetrics and Gynaecology, Bakırköy Sadikonuk Education and Research Hospital, Istanbul, TUR; 2 Obstetrics and Gynaecology, Bakırköy Sadi Konuk Education and Research Hospital, Istanbul, TUR; 3 Obstetrics and Gynaecology, Bağcılar Education and Research Hospital, Istanbul, TUR

**Keywords:** umblical insertion, safety, primary trocar insertion, laparoscopy, adhesions

## Abstract

Introduction: This study aimed to assess the safety of laparoscopic entry sites in patients with previous abdominal surgery who subsequently required re-operation.

Material and methods: This is a prospective study wherein the data of 118 patients who had undergone previous abdominal surgery and were subsequently re-operated at our center (Bakırköy Doctor Sadi Konuk Research and Study Hospital) were collected from October 2015 to October 2016. Careful attention was paid to gathering information regarding patients' age, parity, body mass index (BMI), type of previous surgery, type of incision made during previous surgery, and medical history. For this study, the abdomen was topographically divided into nine parts. During the operation, all quadrants were examined and evaluated for adhesion and the content of adhesion.

Results: Adhesions were found in 44% (55 out of 118) of the patients, while 56% (66 patients) had no adhesions in the abdomen. The majority of cases (74%) had a history of cesarean section, and 87% had a Pfannenstiel incision. Adhesions were reported in 37.5% (33 out of 88) of the patients with a previous history of cesarean section. A significant proportion of subjects with adhesion (83%) had anterior abdominal wall adhesions, including only the omentum, whereas 11.5% (six subjects) had umbilical adhesions. Subjects with a history of umbilical hernia repair had more adhesions.

Discussion: The present study sought to assess the safety of laparoscopic entry points in individuals with prior abdominal surgery. The rise in laparoscopic surgeries, favored for reduced wound infections and quicker recovery times, brings forth concerns about potential complications in those with previous abdominal operations. Historically, postoperative adhesions have been observed in a significant number of patients after gynecological procedures. Our research, however, found a lower adhesion rate, which could be due to the smaller size of our sample and fewer gynecological cases. Existing adhesions can complicate subsequent surgeries, increasing operational times and posing injury risks. Adhesions also elevate healthcare costs and patient morbidity and mortality. Moreover, complications like Trocar-related injuries, including damage to major organs, are pivotal. While certain trocar insertion techniques may have fewer complications, our results align with previous findings suggesting higher adhesion rates after non-gynecological surgeries. Therefore, alternative entry points or methods, such as the palmer site or direct trocar entry, are recommended for those with an abdominal surgery history. Notably, our study's limited sample size may affect its generalizability, urging future studies for broader insights. Comprehensive pre-surgery assessments are crucial to anticipate complications. Our research supports that laparoscopic surgeries are safe for many with prior abdominal surgery, but for certain patients, non-umbilical entry sites are advised to further mitigate risks.

Conclusion: The umbilicus is one of the safest entry sites for primary trocar insertion in patients with a history of Pfannenstiel incision. However, the probability of umbilical adhesions is high in patients who have undergone umbilical mesh repair, median incision, or major abdominal surgery. In these patients, surgeons should prefer other laparoscopic entry sites, especially Palmer's point, rather than the umbilicus.

## Introduction

Laparoscopic surgery has been lauded for its minimally invasive nature, causing less postoperative pain, reduced recovery time, and lower risk of wound complications than open surgery [1]. However, as the practice expands and becomes more commonplace, it's crucial to consider potential challenges. One such challenge is the safety of laparoscopic entry points in patients with previous abdominal surgery.

Historically, open abdominal surgery often leads to developing intra-abdominal adhesions - fibrous bands that form between tissues and organs [2]. These adhesions can pose significant risks during subsequent surgeries, precisely laparoscopic procedures, due to potential injury to the underlying adhered organs during trocar insertion [3]. Consequently, selecting various laparoscopic entry sites sparks interest and discussion regarding safety and achieving the best results for patients.

Traditionally, the primary entry point for laparoscopy is the umbilicus, given its central location and the reduced likelihood of major blood vessels in the vicinity [4]. However, in patients with a history of certain types of abdominal surgery, particularly those involving the umbilical area or midline incisions, the risk of adhesions is increased. This raises the question of whether alternative laparoscopic entry points could provide safer options for this patient population.

Palmer's point, located in the left upper quadrant of the abdomen, has been suggested as a safer alternative entry point, especially in patients with a history of lower abdominal surgeries. It's thought that this site is less likely to be involved in adhesions following such surgeries, hence posing a lesser risk of organ injury during trocar insertion [5].

This article discusses the presence of a safe area where the Veress needle or the initial trocar can enter the abdominal cavity without injuring underlying organs or vessels during various laparoscopic procedures, especially in patients with a history of abdominal surgery. This safety zone can vary depending on anatomical characteristics, the patient's past surgical history, and the technique applied. It will provide a comprehensive review of the existing literature, identify gaps in our knowledge, and suggest directions for future research.

## Materials and methods

The research was done between October 2015 and October 2016, encompassing 118 patients with prior abdominal surgeries. The goal was to assess the safety of laparoscopic entry sites in later gynecological surgical procedures. The safety status at laparoscopic entry sites is commonly referred to as "laparoscopic entry safety" or "laparoscopic entry safety zone/area." This refers to the safe region where, during laparoscopic surgery, the Veress needle or initial trocar can be inserted into the abdominal cavity without injuring underlying organs or vessels. This safety zone can vary based on anatomical features, the patient's past surgical history, and the technique used. Participants were included if they were between the ages of 18 and 55. Individuals with coagulation disorders or connective tissue diseases were excluded from the study.

Information regarding the patient's age, parity, body mass index, surgical and medical history, type of surgery (laparoscopic or laparotomic), the incision technique applied in previous surgeries, and any associated complications were extracted meticulously from their medical files and operation records. The data collection for this study was conducted using two primary methods. First, we utilized an anamnesis form, which allowed patients to provide detailed accounts of their medical history. This form ensured that we had comprehensive information about each patient's health background. Second, we employed a computer data system. This sophisticated system enabled us to store, organize, and analyze the collected data efficiently. By leveraging these two tools, we aimed to achieve a thorough and systematic data collection process, ensuring accuracy and comprehensiveness in our research.

Previous surgical incisions were classified into several types, including Pfannenstiel incision, median incision, transverse umbilical incision, subcostal incision, McBurney incision, and those resulting from laparoscopic procedures. Procedures that were included within the scope of this study ranged from those dealing with minimal adhesions (as evaluated by the surgeon), early-stage endometriosis ablation, ovarian biopsy and puncture, treatment of ectopic pregnancy and pelvic inflammatory disease (including pelvic abscess), cystectomy, salpingectomy, oophorectomy, to more extensive procedures such as adhesiolysis, hysterectomy, myomectomy, colposuspension, and lymphadenectomy in malignant cases. This article is derived from the author Ender Güven's thesis completed in 2016.

Statistical analysis

In analyzing the collected data, descriptive statistical metrics, including means, standard deviations, medians, minimum and maximum values, as well as frequencies and ratios, were utilized. The Kolmogorov-Smirnov test was employed to determine the distribution of the variables. For the analysis of numerical data, both the Mann-Whitney U test and the independent sample t-test were utilized, depending on the data's distribution. On the other hand, for categorical data analysis, the Chi-square test was used. When the conditions for the Chi-square test were not met, Fisher's exact test was applied instead. The statistical software SPSS version 22.0 (IBM Corp., Armonk, NY) served as the primary tool in executing these analyses.

## Results

The demographic features of the patients are presented in Table 1. The average age of the participants in this study was 43, while the mean parity was 2 (Table 1).

**Table 1:** Demographic Information

	Min-Max	Median	Mean.±s.
Age	16-78	43.0	43.1±10.2
Height	150-175	160.0	161.1±4.9
Weight	48-130	67.0	69.3±11.5
Gravida	0-9	2.0	2±1.5
Parity	0-6	2.0	2.1±1.1

The demographic characteristics of the study population are detailed in Table 1. Within the pool of 118 patients who had prior surgical procedures, intra-abdominal adhesions were identified in 52 patients, accounting for 44% of the total, while no adhesions were found in the remaining 66 patients, or 56% of the group. A considerable majority (83%) of the patients in the adhesion group had omental adhesions located on the anterior abdominal wall. Furthermore, seven out of the nine remaining patients exhibited omental adhesions in the paracolic region. Two patients presented widespread omental and intestinal adhesions throughout the abdomen subsequent to gastric resection and Whipple procedures. Adhesions in the umbilical region were found in six patients, amounting to 11.5% of the group with adhesions. Given the total of 118 patients, it was determined that in 95% of cases, no adhesions were present in the umbilical region, which is the most commonly utilized main trocar entry site (Table 2).

**Table 2:** Anatomical Distribution and Content of Adhesions

		n	%
Intra-Abdominal Adhesion	(-)	66	55.90%
	(+)	52	44.10%
Omental adhesion in anterior wall		43	36.40%
Left paracolic omental adhesion		4	3.40%
Right paracolic omental adhesion		3	2.50%
Diffuse bowel and omental adhesion		2	1.70%
Adhesion at the Main Trocar Entry Point	(-)	112	94.90%
	(+)	6	5.10%

A majority of the patients (74%) had a history of cesarean section, and 87% had a Pfannenstiel incision. Six patients had subcostal incisions, and three had median incisions, while five patients had peri-umbilical incisions due to prior umbilical hernia repairs. A Veress needle was used to access the abdomen in 84 patients: three through the epigastric region, three via Palmer's point, and 78 through the umbilicus. Direct laparotomy was performed in 31 patients, while the Hasson technique was utilized in three cases. No complications were encountered during laparoscopic or laparotomic abdominal insertions (Table 3).

**Table 3:** Percentage Distribution of Operation History, Old Incision Type and Abdominal Entry Sites

		n	%
Operational History			
C-Section		87	73.70%
Laparoscopic cystectomy		10	8.50%
Laparatomic cystectomy		21	17.00%
Myomectomy		9	7.60%
Umbilical Hernia Repair		5	4.20%
Appendectomy		11	9.30%
Cholecystectomy		7	5.90%
Total Number of Abdominal Surgery	I	54	45.80%
	II	50	42.40%
	III	12	10.20%
	IV	2	1.70%
Old Incision Type			
Pfannenstiel		103	87.30%
Median Incision		3	2.50%
Veres from the umbilicus		12	10.20%
Transverse Umbilical		5	4.20%
Subcostal		6	5.10%
Mc Burney		8	6.80%
Hypertrophic Scar Or Keloid	(-)	114	96.60%
	(+)	4	3.40%
Abdomen Enterence Zone			
Epigastric		3	2.50%
Sol Hipocondriac		3	2.50%
Umbilical Zone		104	88.10%
Hypogastric		31	26.30%
Abdomen Enterence Tecnique	Veres	84	71.20%
	Laparotomy	31	26.30%
	Hasson	3	2.50%

There was no significant difference in the age, height, weight, number of pregnancies (gravida), and number of births (parity) between the group with intra-abdominal adhesions and the group without adhesions (p > 0.05). Furthermore, there were no significant discrepancies in the rates of additional diseases, hypertension, hyperlipidemia, diabetes mellitus, and hypothyroidism between these two groups (p > 0.05) (Table 4).

**Table 4:** Additional Disease

Additional Disease	n	%
None	81	68.6%
Yes	37	31.4%
Hypertension	21	17.8%
Hyperlipidemia	3	2.5%
Diabetes Mellitus	21	17.8%
Hypothyroidism	2	1.7%

Among the 88 patients with a history of cesarean sections, we identified adhesions in 33 cases, representing a 37.5% incidence rate. Furthermore, our data revealed a significant correlation between an increase in the number of previous abdominal surgeries and a heightened rate of intra-abdominal adhesions (Tables 5-6).

**Table 5:** Intra-abdominal Adhesion Status

	Intra abdominal				
	Adhesion (-)		Adhesion (+)		
	Mean.±s.s	Med	Mean.±s.s	Med	P
Age	42.3±10.9	43	44.1±9.2	43	0.417
Height	160.8±4.7	160	161.5±5.1	160	0.307
Weight	67.8±9.7	65	71.3±13.3	70	0.124
Gravida	2.01±1.1	2	2.5±1.9	2	0.341
Parity	2±1	2	2.2±1.3	2	0.478
Additional Disease	n	%	n	%	
None	49	74.20%	32	61.50%	0.14
Yes	17	25.80%	20	38.50%	
HT	10	15.20%	11	21.20%	0.397
Hyperlipidemia	3	4.50%	0	0.00%	0.254
DM	9	13.60%	12	23.10%	0.183
Hypothyroidism	1	1.50%	1	1.90%	

**Table 6:** Operation History and Adhesion Rates

		Adhesion (-)		Adhesion (+)		
		n	%	n	%	
Operation History						
C-Section		54	81.80%	33	63.50%	0.024
Laparoscopic Cystectomy		4	6.10%	6	11.50%	0.289
Laparatomic Cystectomy		9	13.60%	12	23.10%	0.183
Myomectomy		3	4.50%	6	11.50%	0.155
Umbilical Hernia Repair		1	1.50%	4	7.70%	0.098
Appendectomy		3	4.50%	8	15.40%	0.044
Cholecystectomy		0	0.00%	7	13.50%	0.002
Total Number of Abdominal Surgery Past	I	37	56.10%	17	32.70%	
	II	25	37.90%	25	48.10%	
	III	4	6.10%	8	15.40%	0.028
	IV	0	0.00%	2	3.80%	

The presence or absence of intra-abdominal adhesions did not significantly impact the rates of Pfannenstiel, median, umbilical Veres, transverse umbilical, or McBurney incisions (p > 0.05). Upon examining adhesion status relative to the types of incisions, those with subcostal incisions demonstrated an elevated prevalence of adhesions. Despite lacking statistical significance, all three cases with median incisions, as well as, four out of the five cases involving umbilical hernia sites exhibited intra-abdominal adhesions (Table 7).

**Table 7:** Old Incision Types and Adhesion Relationship

	Intraabdominal				
Presence of Adhesion	Adhesion (-)		Adhesion (+)		P
Old Incision Type	n	%	n	%	
Pfannenstiel	61	92.40%	42	80.80%	0.059
Median incision	0	0.00%	3	5.80%	0.083
Veres from the umbilicus	4	6.10%	8	15.40%	0.096
Transverse Umblical	1	1.50%	4	7.70%	0.098
Subcostal	0	0.00%	6	11.50%	0.005
Mc Burney	3	4.50%	5	9.60%	0.277

There was no significant difference in the rates of hypertrophic scars or keloids between the groups with and without intra-abdominal adhesions (p > 0.05) (Table 8).

**Table 8:** Relationship Between Hypertrophic Scar or Keloid and Intra-Abdominal Adhesion

Location		Intra Abdominal				
Presence of Adhesion		Adhesion (-)		Adhesion (+)		
Ratio		n	%	n	%	P
Hypertrophic Scar Or Keloid	(-)	65	98.50%	49	94.20%	0.319
	(+)	1	1.50%	3	5.80%	

The presence or absence of intra-abdominal adhesions did not significantly influence the rates of epigastric, left hypochondriac, umbilical, or hypogastric insertion sites (p > 0.05). Moreover, there were no significant differences in the rates of Veress, laparotomy, or Hasson techniques between the groups with and without adhesions (p > 0.05) (Table 9).

**Table 9:** Abdominal Entrance Area and Adhesion Relationship

	intraabdominal				
Availability of Adhesion	Adhesion	(-)	Adhesion	(+)	P
Abdominal Enterence Ratio	n	%	n	%	
Epigastric	2	3.00%	1	1.90%	1
Sol Hipocondriac	0	0.00%	3	5.80%	0.083
Umbilicus	59	89.40%	45	86.50%	0.634
Hipogastric	19	28,80%	12	23.10%	0.484
Abdominal Entry Technique Ratio	n	%	n	%	P
Veres	46	69.70%	38	73.10%	0.843
Laparotomy	19	28.80%	12	23.10%	0.624
Hasson	1	1.50%	2	3.80%	0.582

## Discussion

The findings of this study shed light on an important clinical question regarding the potential risks and complications associated with laparoscopic entry in patients with prior abdominal surgical interventions [6]. The prevalence of laparoscopic surgical interventions has shown a notable rise among adult and pediatric populations in recent years. This surgery method presents several advantages, including a significant decrease in the incidence of wound infections, accelerated postoperative recovery times, which promote the quicker resumption of routine activities, and diminished demand for postoperative analgesics [7]. Following gynecological operations, postoperative adhesions have been reported in approximately 70-95% of cases [2]. An investigation involving 624 subjects with a history of abdominal surgery revealed intra-abdominal adhesions in 487 patients (approximately 78%), and umbilical adhesions were identified in 404 patients (approximately 64.7%) [8].

In contrast, our research discovered that 66 patients (comprising 55.9% of the study group) displayed no evidence of intra-abdominal adhesion, whereas adhesions were present in 52 patients (constituting 44.1%). The reduced adhesion rate observed in our research, compared to previous studies, could be due to the smaller sample size of our group and the decreased percentage of gynecological cases in the specific subgroup [9,10]. The existence of adhesions resulting from prior surgical interventions has been observed to extend the duration of subsequent operations by a minimum of fifteen minutes. In conjunction with this, adhesiolysis, or the dissolution of adhesions, has been associated with an elevated risk of injuries to the bowel and other grave complications that can potentially threaten the patient's life [11,12].

In terms of fiscal and health-related implications, adhesions contribute to increased healthcare costs as well as elevated rates of morbidity and mortality. The likelihood of adhesion development following a singular abdominal procedure has been reported to be approximately 47% for appendectomies, which rises to 91% in the case of pelvic surgeries [13].

During laparoscopic procedures, Trocar-related injuries have an important place among possible complications. Major organ damage has been documented at a frequency of 1.1 instances per 1000 laparoscopic access procedures. Of these, intestinal damages were observed at a rate of 0.7 per 1000, while significant vascular injuries were reported at a frequency of 0.4 per 1000 [14].

A comparative analysis of the Veress needle technique and direct trocar insertion procedures in the existing literature revealed that the incidence of major complications in the former was marginally higher. However, it was significantly lower than the incidence associated with the direct trocar insertion method [15]. A separate study contrasted the Hasson trocar insertion technique with the Veress needle method and found no disparity in terms of major complications. Nevertheless, minor complications and unsuccessful insertion attempts were less prevalent in using the Hasson technique [16].

Our findings are consistent with the aforementioned study in the existing literature. Analyzing non-gynecological and gynecological surgical procedures separately yields more dependable and accurate information. Notably, our investigation revealed significantly elevated adhesion rates following non-gynecological surgical procedures. Consequently, for patients with a history of abdominal surgery, it is advisable to employ alternative points of access, such as the Palmer or Jain sites, or alternative entry methods, including the open technique or direct trocar entry [17].

Study limitations

Our study faced certain limitations, chiefly the relatively small sample size which may impact the broad applicability of our findings. This constraint suggests our results might not capture the full array of outcomes related to laparoscopic entry points in patients with prior abdominal surgeries. For a more comprehensive understanding, future research should consider larger participant groups, extended follow-up periods, as well as factors like varying surgical techniques and the surgeon's expertise. This would ensure a holistic assessment of the procedure's safety and long-term effects [18]. 

Potential complications may be anticipated effectively through a comprehensive anamnesis and a meticulous physical examination conducted by a seasoned medical team before the surgical procedure. Based on our research findings, it is reasonable to assert that laparoscopic intervention can be executed safely for a significant proportion of patients with a history of previous abdominal surgery. Further, our data suggest that for most of these patients, the umbilical region represents a safe entry point.

However, we advocate using non-umbilical entry sites for patients who have undergone significant abdominal surgery or those with a history of a median incision or umbilical hernia. This strategy aims to minimize further the potential risk associated with laparoscopic procedures.

## Conclusions

Our research delved into the intricacies of laparoscopic entry points, especially for patients with a history of abdominal surgeries. While the popularity of laparoscopic procedures continues to grow due to their inherent advantages, concerns around postoperative adhesions remain prominent. We observed a relatively lower adhesion rate than previous studies, a deviation possibly stemming from our study's modest cohort size and composition. Importantly, non-gynecological surgeries in our study revealed a more pronounced rate of adhesions, suggesting a nuanced approach when deciding laparoscopic access points for different surgical backgrounds.

Drawing from our findings, it is prudent to highlight the significance of selecting appropriate laparoscopic access points or techniques. Although the umbilical region often emerges as a viable access point, we advocate for alternative sites for patients with extensive abdominal surgeries or specific prior incisions. This tailored approach seeks to optimize the safety and efficacy of laparoscopic interventions, ensuring the best possible outcomes for patients.
